# Protective effects of Notoginsenoside R2 on reducing lipid accumulation and mitochondrial dysfunction in diabetic nephropathy through regulation of c-Src

**DOI:** 10.1186/s13020-024-01057-y

**Published:** 2025-01-15

**Authors:** Xieyi Guo, Liu Yang, Xiaoning An, Maofang Hu, Yilan Shen, Niansong Wang, Youhua Xu, Dingkun Gui

**Affiliations:** 1 Department of Nephrology, Shanghai Sixth People’s Hospital Affiliated to Shanghai Jiao Tong University School of Medicine, Shanghai, China; 2 Graduate School of Jiangxi University of Chinese Medicine, Nanchang, China; 3 Faculty of Chinese Medicine, State Key Laboratory of Quality Research in Chinese Medicine, Macau University of Science and Technology, Taipa, Macao China

**Keywords:** Notoginsenoside R2, Diabetic nephropathy, c-Src, Lipid accumulation, Mitochondrial dysfunction

## Abstract

**Background:**

The treatment options to delay the progression of diabetic nephropathy (DN), a key contributor to chronic kidney disease (CKD), are urgently needed. Previous studies reported that traditional Chinese medicine Panax notoginseng (PNG) exerted beneficial effects on DN. However, the renoprotective effects of Notoginsenoside R2 (NR2), an active component of PNG, on DN have not been investigated. This study aimed to assess the therapeutic potential of NR2 in DN and explore its underlying mechanisms.

**Methods:**

*In vivo* models were developed using db/db mice, while in vitro models utilized HK-2 cells exposed to high glucose and palmitic acid (HGPA). Online databases and Cytoscape software were employed to predict the potential targets of NR2. The expression of associated proteins was measured using immunohistochemistry and western blot. Lipid accumulation, oxidative stress levels, mitochondrial function and cell apoptosis were also assessed. Small interfering RNA was used in in vitro experiments to examine the effect of c-Src.

**Results:**

NR2 ameliorated albuminuria, renal function and renal pathology in db/db mice. The activation of c-Src was suppressed in db/db mice and in HK-2 cells exposed to HGPA. NR2 inhibited JNK/STAT1 phosphorylation and CD36 overexpression. NR2 also ameliorated lipid accumulation, oxidative stress, mitochondrial dysfunction and cell apoptosis in vivo and in vitro. By inhibiting c-Src, HK-2 cells exposed to HGPA experienced less lipid deposition and mitochondrial damage, indicating the renoprotective effects of NR2 were correlated with the inhibition of c-Src.

**Conclusion:**

NR2 ameliorated mitochondrial dysfunction and delayed the progression of DN partly through suppression of c-Src. The protective effects of NR2 might be related to a reduction in lipid accumulation.

**Graphical Abstract:**

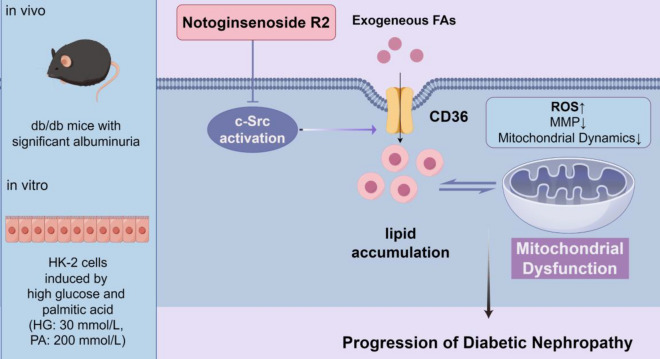

## Introduction

Chronic kidney disease (CKD) is mainly attributed to diabetic nephropathy (DN). Diabetic individuals are estimated to develop DN at an approximate rate of 40%, with 50% of these cases advancing to end-stage renal disease (ESRD) [1, 2]. Therefore, it is urgent to develop innovative interventions to delay the progression of DN. Lipids are essential for a wide range of cellular functions and are integral to maintaining cellular homeostasis. However, excessive lipid accumulation disrupts homeostasis, leading to cell dysfunction and ultimately triggering cell death [3]. The patients with DN are usually accompanied by dyslipidemia and ectopic lipid deposition [4]. The first stage of lipid metabolism is represented by the absorption of fatty acids. The cluster of differentiation 36 (CD36) protein plays a pivotal role in fatty acid uptake, as confirmed by previous studies [5, 6]. Hyperglycemia has been shown to upregulate CD36 expression, thereby promoting fatty acid uptake and exacerbating lipid accumulation [7]. Inhibiting CD36 activity could restrict the mobilization of fatty acids from adipose tissue to non-adipose tissue [8]. Elevated levels of CD36 are associated with an increased production of reactive oxygen species (ROS), which in turn leads to mitochondrial dysfunction [9]. Targeting CD36 might represent a novel approach to prevent the progression of DN.

Recent studies demonstrated that elevated levels of fatty acids alone were insufficient to induce cellular disorders, suggesting that mitochondrial damage might also contribute to lipid deposition-induced cellular harm [10]. ROS disrupted metabolic pathways by compromising the stability and activity of various metabolic enzymes [11, 12]. Additionally, ROS inhibited oxygen consumption in adipocytes, leading to ectopic lipid accumulation [13]. Evidence indicated that mitochondrial protection and ROS mitigation might prevent renal dysfunction by normalizing dysregulated lipid metabolism. Reducing mitochondrial oxidative stress decreased lipid deposition, alleviated lipid peroxidation, and mitigated cellular damage [14].

Panax notoginseng saponins (PNS), the active components of the traditional Chinese medicine Panax notoginseng (also known as Sanqi). Previous studies demonstrated that PNS effectively delayed the progression of kidney diseases [15, 16]. PNS contains multiple saponins including Notoginsenoside R1, Notoginsenoside R2, Ginsenoside Re, Ginsenoside Rg1, Notoginsenoside Fc and so on. Our previous study demonstrated that Notoginsenoside R1 improved podocyte adhesion under diabetic circumstances by upregulating α_3_β_1_ integrin [17] and Notoginsenoside Fc alleviated pyroptosis in glomerular endothelial cells by inhibiting the HMGCS2 pathway [18]. Ginsenoside Rg1 and Ginsenoside Re inhibited oxidative stress and reduced renal fibrosis, thereby slowing DN progression [19–21]. However, the effects of Notoginsenoside R2 (NR2), a novel saponin isolated from Panax notoginseng, on DN have not been investigated. This study aimed to assess the therapeutic potential of NR2 in DN and explore its underlying mechanisms.

## Methods

### Chemicals and reagents preparation

Notoginsenoside R2 (HPLC purity: ≥ 98%) was purchased from DeSiTe Biological Technology Co., Ltd. (Chengdu, China). Losartan was from Merck Sharp and Dohme Ltd. (USA). The test kits for measuring creatinine, urea nitrogen, cholesterol, triglycerides (TG), glucose, alanine transaminase (ALT), aspartate transaminase (AST), malondialdehyde (MDA), superoxide dismutase (SOD), glutathione (GSH), catalase (CAT), ATP, and ELISA kits for mouse urine microalbumin were purchased from Nanjing Jiancheng Bioengineering Institute (Nanjing, China). Glutathione Reductase (GR), JC-1, TUNEL detection, and antibody p-Tyr416 Src (AF5923) were purchased from Beyotime. Staining kit of Oil Red O was purchased from Solarbio. BODIPY fluorescent dye was supplied by MedChemExpress. MitoSOX Red Mitochondrial Superoxide Indicator was obtained from Thermofisher. Annexin V-FITC/PI Apoptosis Detection Kit was purchased from BD Biosciences. A small interfering RNA (siRNA) for c-Src was obtained from Ribobio Biotechnology (Guangzhou, China). Antibodies including p-Tyr416 Src (2101), Src (2123), p-JNK (9252), JNK (9251), p-STAT1 (7649), STAT1 (9172), Drp1 (8750), Mfn2 (9482), Fis1 (32525), Bcl-2 (3498), cleaved-caspase-3 (9664) were from CST (USA). CD36 (ab252922) was obtained from Abcam (UK). Bax (50599–2-Ig), Bcl-2 (80313–1-RR), p-JNK (80024–1-RR), JNK (66210–1-Ig), smooth muscle actin (14395–1-AP), Fibronectin (66042–1-Ig), Vimentin (10366–1-AP), β-actin (20536–1-AP) were from Proteintech (Wuhan, China).

### Animal experiments

**Fig. 1:**
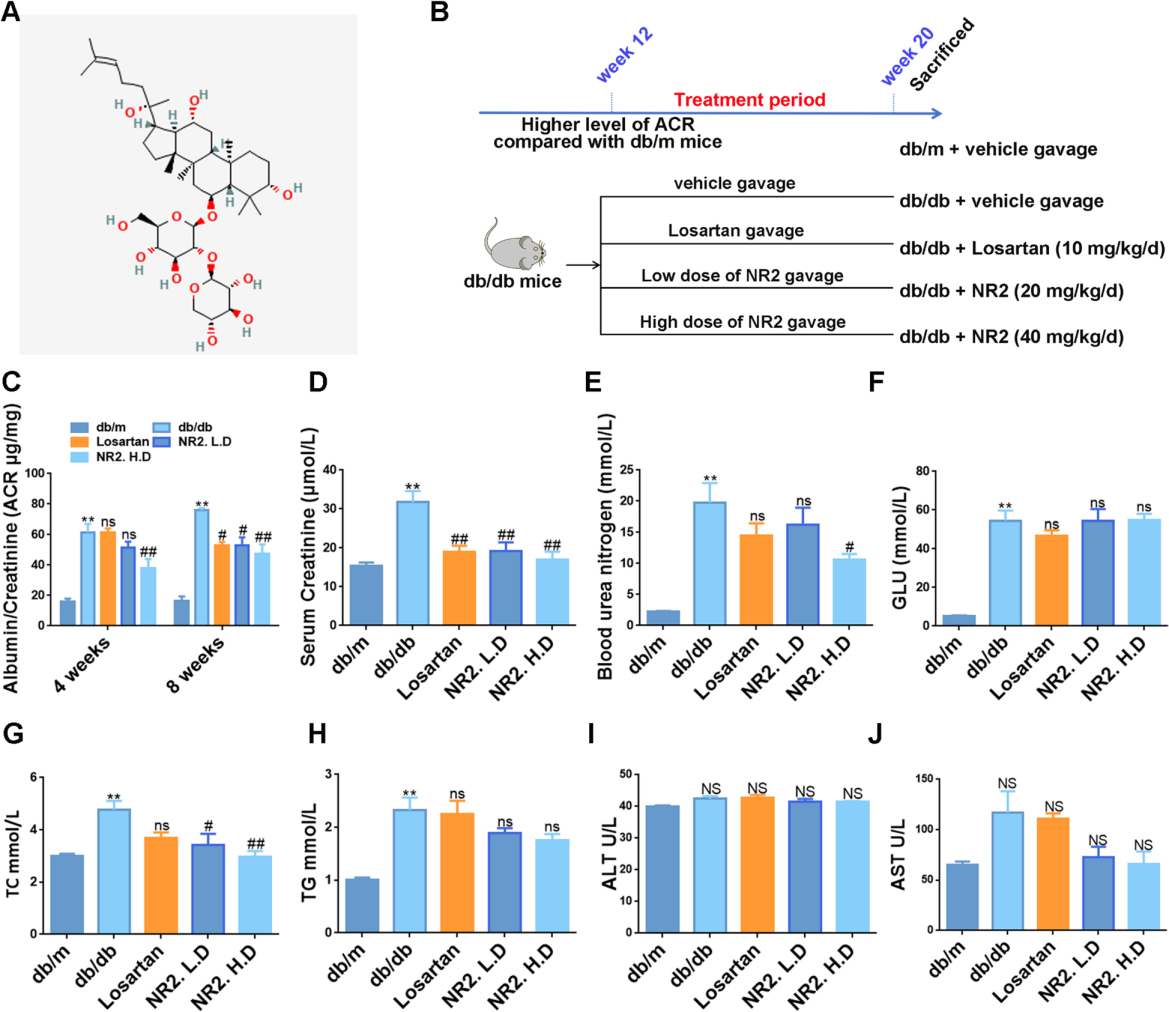
Effects of Notoginsenoside R2 on biochemical characteristics in db/db mice. **A** The chemical structure of Notoginsenoside R2 (Notoginsenoside R2, C_41_H_70_O_13_, Molecular Weight 770.99). **B** Animal experimentation time-series and grouping structure. **C** The levels of ACR in each group of mice at week 4 and 8 (n = 6). **D**–**J** The levels of Scr, BUN, Blood glucose, Serum TC, Serum TG, ALT, and AST (n = 6). (*p < 0.05 and **p < 0.01 compared with db/m mice; ^#^p < 0.05 and ^##^p < 0.01 compared with db/db mice; ns: no statistical difference compared with db/db mice; NS: no statistical difference compared with db/m mice)

The animal experiment protocol was approved by the Animal Ethics Committee of Shanghai Sixth People’s Hospital Affiliated to Shanghai Jiao Tong University School of Medicine. Male C57BKS/Lepr db/db mice aged 7–8 weeks and age-matched db/m controls were obtained from Ziyuan Experimental Animal Technology Co., Ltd. (Hangzhou, China). Temperature-controlled environments were prepared for experimental mice with ad libitum access to food and water in a 12-h cycle of light and dark. After approximately 12 weeks, urine albumin-to-creatinine ratios (ACR) were measured. When db/db mice exhibited significantly elevated ACR levels, they were randomly assigned to the following groups: the model group (db/db), the group receiving Losartan (10 mg/kg/day), the low-dose Notoginsenoside R2 group (20 mg/kg/day, NR2. L.D) and the high-dose Notoginsenoside R2 group (40 mg/kg/day, NR2. H.D). Losartan was selected as a positive control based on previous study [22]. The dosages of NR2 were selected according to the published literature [23] and our preliminary experiment. The db/m mice were used as the standard control group (db/m). Each group consisted of 6 mice and Losartan or Notoginsenoside R2 were administered daily via gavage for 8 weeks. During the experiment, db/m and db/db models received equivalent volumes of saline via gavage. The summary of the animal experimental protocol is illustrated in Fig. 1B. Following the 8-week treatment period, the mice were anesthetized with the use of isoflurane and subsequently euthanized.

### Measurement of urine and blood biochemical parameters

Urinary microalbumin and creatinine levels were quantified from the supernatant of urine samples that were centrifuged at 3000 rpm for 15 min. Urinary albumin excretion was assessed through the albumin-to-creatinine ratio (ACR). Blood samples were collected post-sacrifice, and serum creatinine (Scr), blood urea nitrogen (BUN), blood glucose, total cholesterol (TC), triglycerides (TG), alanine aminotransferase (ALT), and aspartate aminotransferase (AST) levels were determined using commercial assay kits.

### Renal histological analyses

Kidney samples were embedded in paraffin wax following fixation in 4% paraformaldehyde. Subsequent to deparaffinization, the samples underwent staining with Hematoxylin and Eosin (HE), Periodic Acid-Schiff (PAS) and Masson’s trichrome stains. Light microscopy was then employed to inspect the stained slides.

### Transmission electron microscopy (TEM) studies

Freshly prepared 2.5% glutaraldehyde was utilized for primary fixation. Subsequently, 1 mm kidney tissue samples were rinsed and subjected to post-fixation using 1% osmium tetroxide. After a graded ethanol dehydration series, tissues were embedded in epoxy resin. Ultrathin sections were stained with lead citrate and 2% uranyl acetate and images were obtained using a TEM at 80 kV.

### The screen of the potential target of Notoginsenoside R2

PharmaMapper (http://lilabecust.cn/pharmmapper/submitfile.html) was employed to search for the potential targets of Notoginsenoside R2 and GeneCards (http://www.genecards.org) was applied to identify the targets associated with DN. The construction and visualization of a protein–protein interaction (PPI) network were performed using the STRING database in conjunction with Cytoscape software to identify core targets. The DAVID online platform was subsequently applied to perform pathway enrichment analysis, revealing potential therapeutic targets.

### Immunohistochemistry (IHC) staining

IHC staining was performed to evaluate the expression of CD36 and phosphorylated c-Src (p-Tyr416) in kidney tissue samples. Tissue sections were cut into 4 μm slices and dewaxed using a graded ethanol series. Following the inhibition of endogenous peroxidase activity with 0.3% hydrogen peroxide for 15 min, the tissue sections were incubated in 5% bovine serum albumin (BSA) for 1 h to minimize nonspecific binding. Then, for incubation, pre-diluted primary antibodies against CD36 and p-Tyr416 c-Src were added. Incubation at 4 °C for the entire night before being subjected to the secondary antibodies. Finally, the sections were counterstained with hematoxylin and developed using diaminobenzidine (DAB) before being examined under a light microscope.

### Assessment of intrarenal lipids

To evaluate lipid accumulation, frozen kidney tissue sections were stained with Oil Red O. After fixation in 4% paraformaldehyde, the sections were immersed in 60% isopropanol to facilitate staining. Meanwhile, a fresh working solution of Oil Red O was prepared. The sections were incubated in the working solution at room temperature for 15 min and then rinsed with isopropanol. After counterstaining and mounting, images were captured under a light microscope.

Kidney tissues were homogenized and supernatants were collected by centrifugation at 12,000 rpm for 15 min. These supernatants were then used to assess TG content using a triglyceride measurement kit.

### Cell intervention

Human renal proximal tubular cells (HK-2) were cultured at 37 °C in DMEM media containing 10% fetal bovine serum with 5% CO2. To simulate type 2 diabetic conditions, cells were treated with high glucose (30 mmol/L) and palmitic acid (200 mmol/L) (HGPA) [24]. For the evaluation of NR2 effects, cells at the confluence of 80% were randomly divided into 6 groups: normal control group (Ctr), high glucose and palmitic acid stimulation group (HGPA), Losartan group (Losartan, HGPA + 50 μM Losartan) [22], low dose of NR2 (NR2. L.D, HGPA + 10 μM NR2), medium dose of NR2 (NR2. M.D, HGPA + 20 μM NR2), and high dose of NR2 (NR2. H.D, HGPA + 40 μM NR2) and then incubated for 24 h.

### Estimation of lipid peroxide and antioxidant indexes

The broken-up kidney tissues or HK-2 cells were centrifuged to get the supernatants, which were used to measure antioxidant parameters. Commercial assay kits were used to measure MDA, SOD, GSH, GR, CAT, and ATP levels.

### BODIPY staining

After fixation with 4% paraformaldehyde, the cellular lipids were stained for 30 min with BODIPY dye and the nuclei were counterstained using DAPI. Images were captured using a fluorescence microscope.

### ROS detection

Mitochondrial superoxide generation was assessed using MitoSOX Red indicator. A 10 μM MitoSOX dye solution was prepared and used to incubate living HK-2 cells for 20 min. Hoechst 33,342 (Beyotime) was applied to counterstain nuclei and the images were recorded by fluorescence microscope.

### TUNEL assay

Frozen tissue sections were fixed and permeabilized, after which a TUNEL detection solution was applied. The sections were subsequently incubated for 1 h in the dark. Sections were counterstained with DAPI, and a fluorescence microscope was applied to identify apoptotic cells.

### Flow cytometry analysis

Upon trypsinization, HK-2 cells were detached and transferred to flow cytometry tubes. The cell suspensions were centrifuged at 1000 rpm for 5 min, after which the supernatant was discarded. Each tube received 0.5 mL of culture medium to gently resuspend the cell pellet. Subsequently, JC-1 working buffer was introduced, and the cells were incubated at 37 °C for 30 min. Following this incubation, the cells were washed and resuspended in JC-1 buffer, allowing for the assessment of mitochondrial membrane potential using flow cytometry.

For apoptosis detection, detached HK-2 cells were centrifuged and resuspended in pre-warmed culture medium. The labeling reagents FITC and PI were added in succession, and the cells were incubated in the dark at room temperature for 15 min. Flow cytometry analysis was performed after adding binding buffer to the labeled cell suspensions.

### siRNA transfection

HK-2 cells were subjected to a 12-h starvation period upon reaching approximately 50% confluence. The siRNA-c-Src transfection mixture was gently introduced to the culture medium. After 24 h of incubation, cells were then stimulated with HGPA, and NR2 (40 μM) was added into one of the experimental groups. Samples were collected for further analysis 24 h later.

### Western blot analysis (WB)

To prepare kidney samples or cell lysates containing total proteins, RIPA lysis buffer was supplemented with protease and phosphatase inhibitors in a 1:100 dilution. The resulting protein mixtures were subjected to SDS-PAGE for separation, followed by transfer onto PVDF membranes. After blocking, the membranes were incubated overnight at 4 °C with primary antibodies. Subsequently, secondary antibodies were added, and the target proteins were visualized using an ECL solution. Imaging of protein bands was performed using the ChemiDoc Touch Imaging System, and band intensity was quantified with ImageJ software, normalizing each band to β-actin expression. Data were analyzed with GraphPad Prism.

### Statistical analysis

Statistical analyses were carried out using GraphPad Prism 7.0. Data are expressed as mean ± standard error of the mean (SEM). One-way analysis of variance (ANOVA) was employed for comparisons among multiple groups, with statistical significance set at P < 0.05.

## Results

### The effect of Notoginsenoside R2 on biochemical characteristics in db/db mice

Different dosages of Notoginsenoside R2 (Fig. 1A) and Losartan were administered to db/db mice exhibiting elevated ACR levels for 8 weeks (Fig. 1B). ACR levels were assessed at 4 and 8 weeks after the initiation of treatment and the results showed that NR2 significantly lowered ACR levels. Notably, the high dose of NR2 caused a greater reduction compared to Losartan (Fig. 1C). Renal dysfunction was ameliorated by NR2 treatment, as evidenced by ameliorating Scr and BUN levels in high-dose NR2-treated db/db mice compared to the untreated group (Fig. 1D, E). Although db/db mice exhibited increasing levels of blood glucose, the difference between treated and untreated mice was not significant (Fig. 1F). Serum lipids were also analyzed, revealing that TC levels were reduced in the NR2-treated group, while TG levels remained unchanged (Fig. 1G, H). We evaluated ALT and AST levels to assess potential drug toxicity and the results indicated that NR2 did not appear to be toxic to the liver (Fig. 1I, J).

### Notoginsenoside R2 ameliorated renal histopathological impairment in db/db mice

**Fig. 2:**
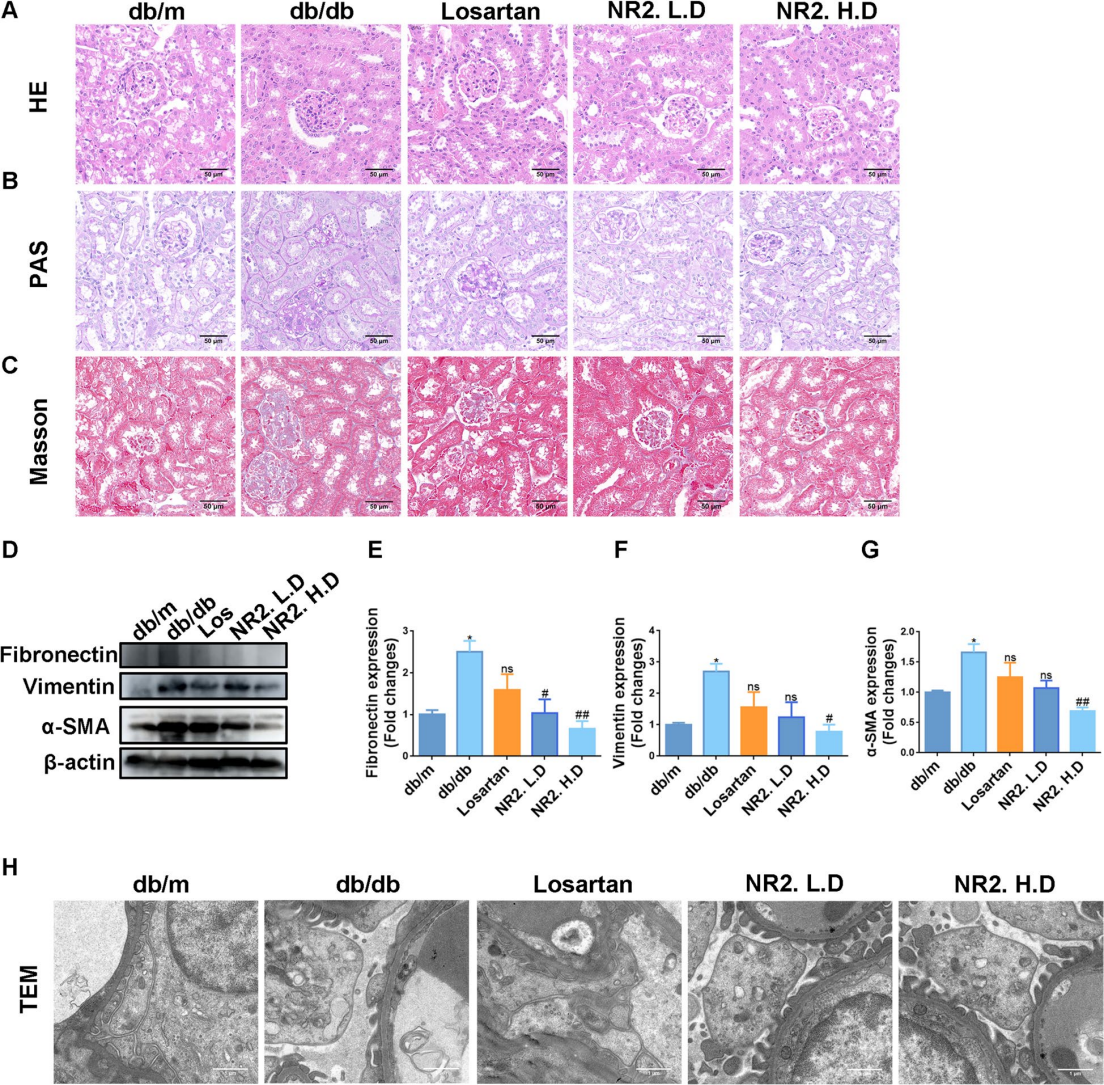
Notoginsenoside R2 relieved histological impairments in db/db mice. **A** Representative HE staining images of kidney sections. **B** Representative PAS staining. **C** Representative Masson staining. **D**–**G** The expression of fibronectin, vimentin, and α-SMA in each group of kidney tissues detected by Western blot and the quantitative analyses (n = 3). **H** Representative TEM images. (*p < 0.05 and **p < 0.01 compared with db/m mice; ^#^p < 0.05 and ^##^p < 0.01 compared with db/db mice; ns: no statistical difference compared with db/db mice)

Histological analyses were conducted to further assess the renoprotective effects of Notoginsenoside R2. HE staining revealed that mesangial expansion, matrix widening, and dilation of renal tubules were present in the model group and these abnormalities were markedly ameliorated in db/db mice treated with Losartan and NR2 (Fig. 2A). In addition to mesangial cell proliferation and renal tubular injury, PAS staining showed glycogen deposition in db/db mice, which was mitigated by NR2 intervention (Fig. 2B). Masson staining suggested that NR2 decreased collagen fiber deposition (Fig. 2C), which was corroborated by WB analysis. A significant reduction in the expression of fibrosis-related proteins, including fibronectin, vimentin, and α-SMA was observed in NR2-treated db/db mice (Fig. 2D–G). Significant podocyte foot process effacement was revealed in TEM images of db/db mice, however, these abnormalities were lessened in mice receiving NR2 treatment (Fig. 2H). Collectively, these findings demonstrated that NR2 exerted a protective effect against kidney histopathological damage associated with DN.

### Notoginsenoside R2 inhibited CD36 expression and might attenuate lipid accumulation by targeting c-Src

**Fig. 3:**
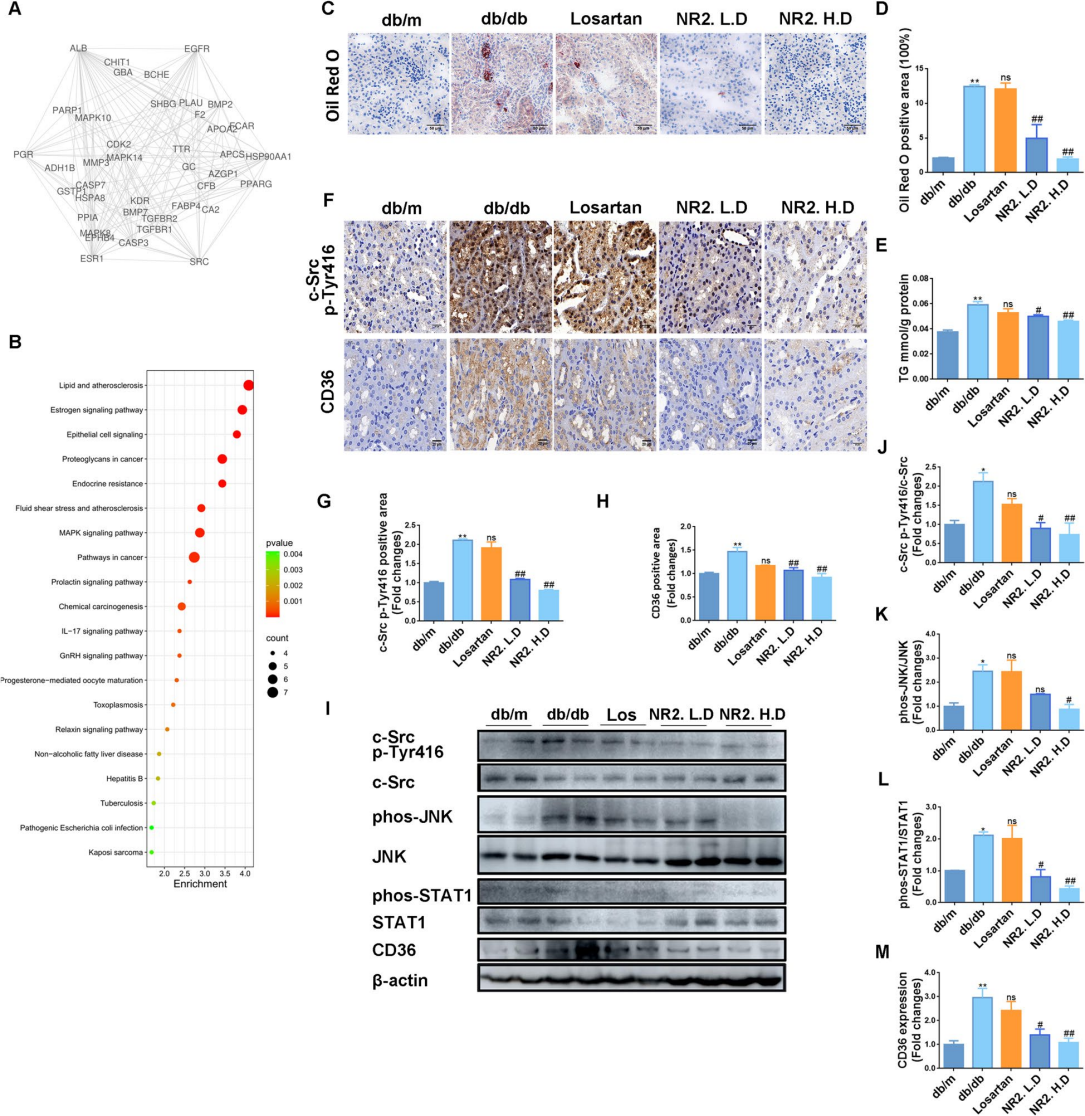
Notoginsenoside R2 ameliorated intrarenal lipids and downregulated CD36 expression by inhibiting c-Src in db/db mice. **A** The protein–protein interaction network of NR2 and DN. **B** The result of KEGG signal enrichment analysis. **C**, **D** Representative images of Oil Red O staining of kidney sections in each group and semiquantitative analysis (n = 3). **E** The triglyceride content in kidney tissues in each group (n = 6). **F–H** IHC images and semiquantitative analysis of c-Src Tyr416 and CD36 (n = 3). **I–M** The expression of phosphorylated c-Src Tyr416, CD36, phosphorylated JNK, and phosphorylated STAT1 in each group of kidney tissues detected by Western blot and the quantitative analyses (n = 3). (*p < 0.05 and **p < 0.01 compared with db/m mice; ^#^p < 0.05 and ^##^p < 0.01 compared with db/db mice; ns: no statistical difference compared with db/db mice)

We further explored the potential mechanisms underlying the renoprotective effects of Notoginsenoside R2. Protein–protein interaction networks and core targets were identified by analyzing the intersecting targets of NR2 and DN (Fig. 3A). These core targets were utilized for Kyoto Encyclopedia of Genes and Genomes (KEGG) pathway enrichment analysis, highlighting that NR2 might influence lipid metabolism pathways (Fig. 3B). To corroborate this prediction, kidney sections from db/db mice were stained with Oil Red O, revealing the presence of lipid droplets, which were significantly reduced by the administration of NR2 (Fig. 3C, D). Measurement of triglyceride content in renal tissue indicated lower levels in NR2-treated db/db mice (Fig. 3E). C-Src, identified as a core target through database predictions (Fig. 3A), was reported to function as an upstream regulator of the fatty acid transporter CD36 via the JNK/STAT1 pathway [25]. The activation of c-Src requires the phosphorylation of the tyrosine site Tyr416. As demonstrated by IHC staining, the overexpression of phosphorylated c-Src was found to decrease in the kidneys of db/db mice upon administering NR2 (Fig. 3F, G). The WB data corroborated the results of the IHC staining (Fig. 3I, J). An apparent increase in CD36 expression was also detected in the db/db group assessed using IHC and WB techniques; however, this increase was significantly suppressed by NR2 treatment (Fig. 3F, H, I, M). Additionally, we investigated the expression levels of phosphorylated JNK (p-JNK) and phosphorylated STAT1 (p-STAT1). The results showed that the activation of p-JNK and p-STAT1 was suppressed by NR2 treatment (Fig. 3I, K, L).

**Fig. 4:**
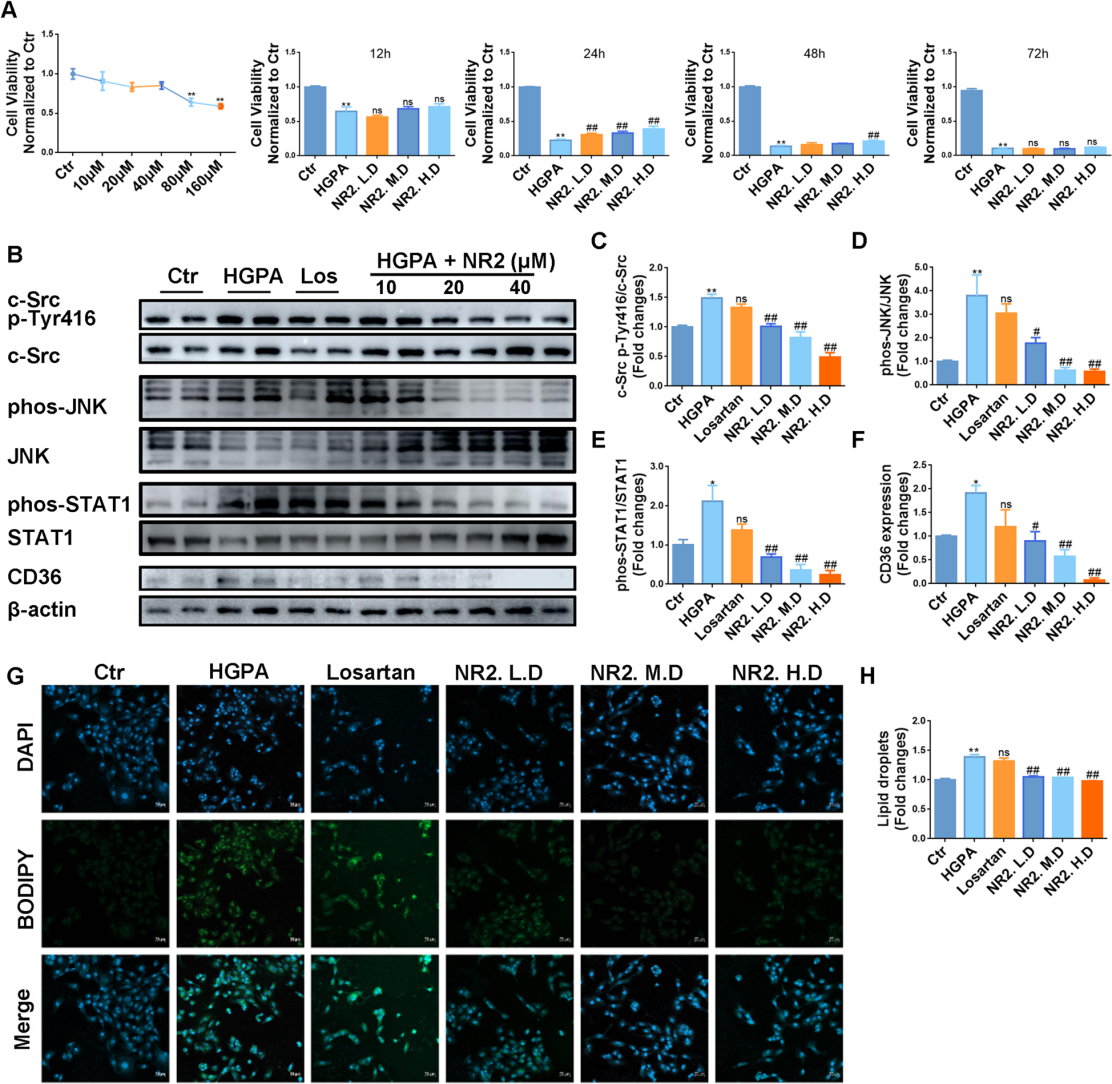
Notoginsenoside R2 ameliorated intracellular lipid droplets and overexpression of CD36 by inhibiting c-Src in cells. **A** MTT experiments to determine the dosage of NR2 and the stimulation time of HGPA. **B**–**F** Western blot analysis for c-Src p-Tyr416, p-JNK, p-STAT1, and CD36 protein expression in HK-2 cells stimulated by HGPA and quantitative analysis (n = 3). **G**, **H** Lipid droplets in HK-2 cells measured by BODIPY staining and semiquantitative analysis (n = 3). (*p < 0.05 and **p < 0.01 compared with normal control group; ^#^p < 0.05 and ^##^p < 0.01 compared with HGPA model group; ns: no statistical difference compared with HGPA group)

Given that proximal tubular cells primarily rely on fatty acids as their energy substrate and are therefore more susceptible to lipotoxicity [26], HK-2 cells were utilized for the in vitro investigations. Through MTT assays, the optimal dosages of NR2 and the duration of HGPA stimulation were determined (Fig. 4A). Following 24 h of HGPA stimulation, WB analysis revealed a significant upregulation of Tyr416-phosphorylated c-Src in the model group, indicating that c-Src activation was dose-dependently inhibited by NR2 treatment (Fig. 4B, C). The overexpression of p-JNK, p-STAT1, and CD36 was alleviated in the NR2-treated group (Fig. 4B, D–F). By using BODIPY staining, HGPA also clearly caused lipid deposition in HK-2 cells, however, these effects were effectively mitigated by NR2 intervention (Fig. 4G, H). Collectively, these results indicated that NR2 attenuated CD36 overexpression via inactivating c-Src and reduced lipid accumulation in vivo and in vitro.

### Notoginsenoside R2 ameliorated oxidative stress and reversed mitochondrial dysfunction

**Fig. 5:**
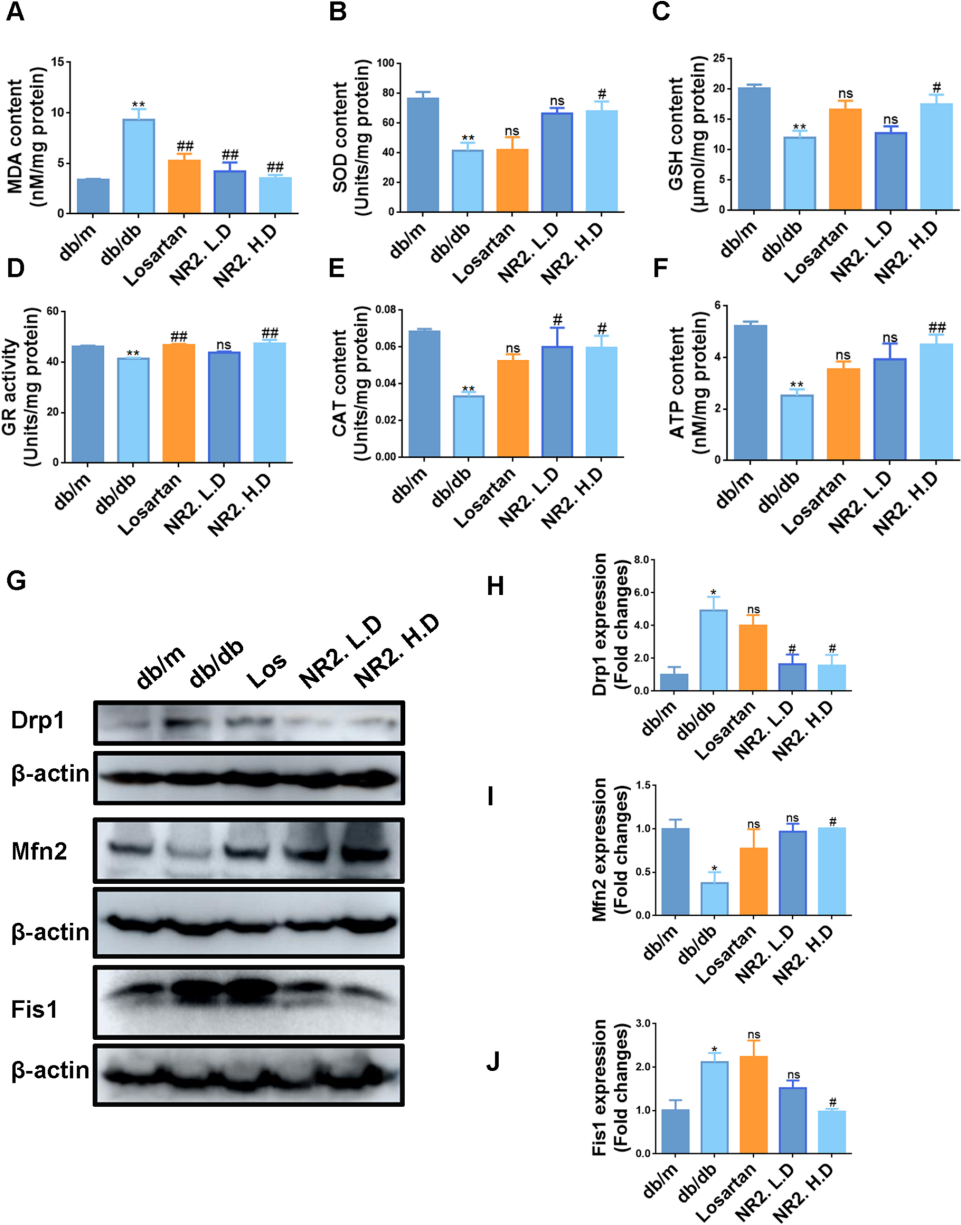
Notoginsenoside R2 attenuated renal oxidative stress level and mitochondrial dysfunction in db/db mice. **A**–**F** The levels of MDA, SOD, GSH, GR activity, CAT, and ATP in kidney tissues of mice (n = 6). **G**–**J** Western blot analysis for Drp1, Mfn2, and Fis1 protein expression in kidney tissues and quantitative analysis (n = 3). (*p < 0.05 and **p < 0.01 compared with db/m mice; ^#^p < 0.05 and ^##^p < 0.01 compared with db/db mice; ns: no statistical difference compared with db/db mice)

Mitochondria are crucial for regulating metabolic disturbances, including alterations in lipid metabolism under diabetic conditions. Disruptions in mitochondrial function led to increased intracellular oxidative stress, affecting lipid metabolic pathways [12]. We next investigated the impact of Notoginsenoside R2 on mitochondrial function. In db/db mice, the MDA level, indicative of lipid peroxidation, was significantly elevated, whereas levels of antioxidant indicators, SOD, GSH, GR, and CAT, were conversely diminished. These impairments were reversed by NR2 administration (Fig. 5A–E). NR2-intervened db/db mice exhibited elevated ATP levels relative to the model group (Fig. 5F). These findings implied that NR2 might alleviate oxidative stress and diminish ROS-induced damage in db/db mice. The expression of Drp1 and Fis1, proteins essential to mitochondrial fission, was also markedly downregulated by NR2 intervention (Fig. 5G, H, J). NR2 also improved the reduction of Mfn2, a protein known for maintaining mitochondrial morphology and function (Fig. 5G, I), highlighting its beneficial effects on mitochondrial function.

**Fig. 6:**
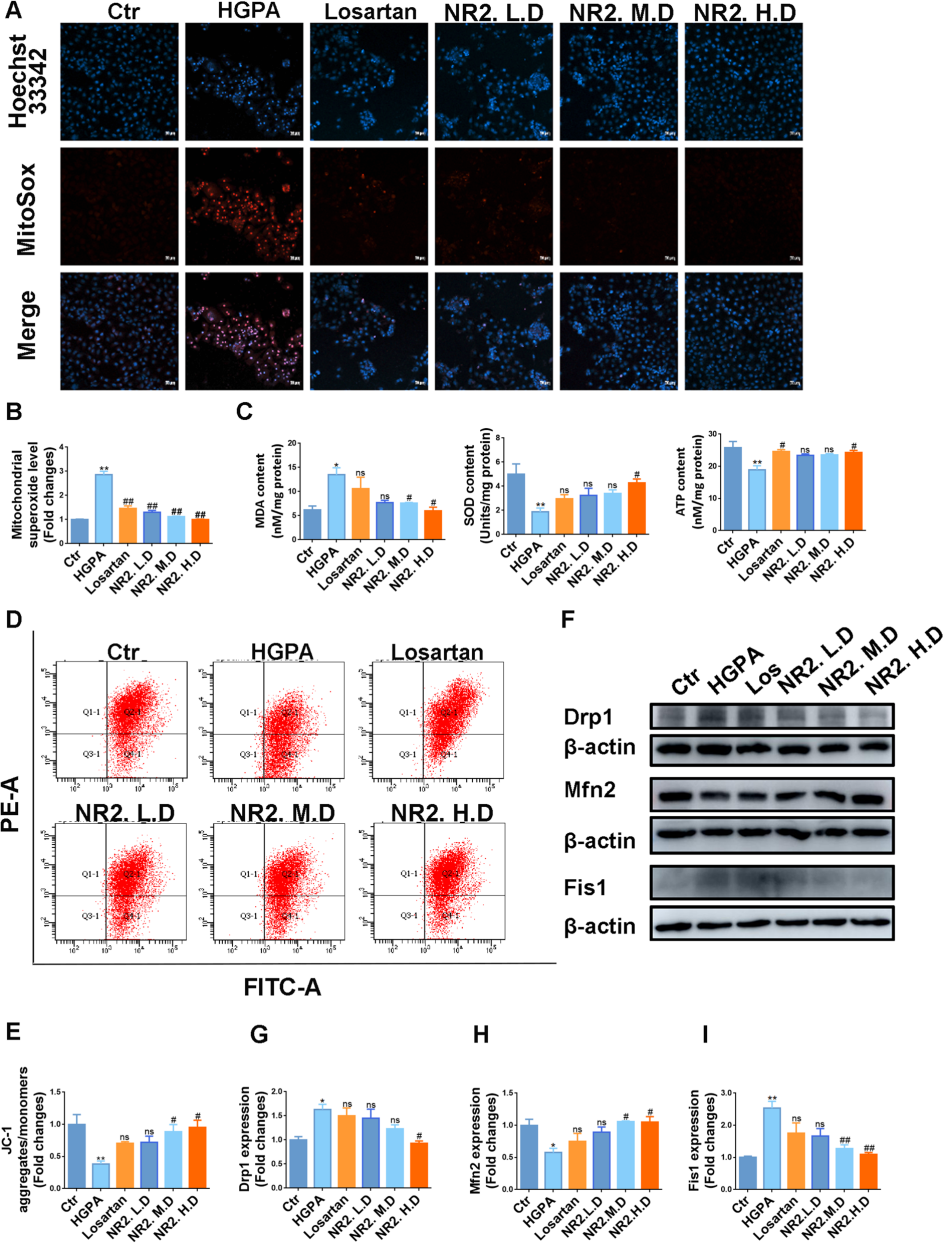
Notoginsenoside R2 attenuated oxidative stress and mitochondrial dysfunction in HGPA-induced HK-2 cells. **A**, **B** Mitochondrial ROS production detected by MitoSOX dye and statistical assessment (n = 3). **C** The levels of MDA, SOD, and ATP in HK-2 cells (n = 3). **D**, **E** The mitochondrial membrane potential of each group HK-2 cells measured by flow cytometry (n = 3). **F**–**I** Western blot analysis for Drp1, Mfn2, and Fis1 protein expression in HK-2 cells and quantitative analysis (n = 3). (*p < 0.05 and **p < 0.01 compared with normal control group; ^#^p < 0.05 and ^##^p < 0.01 compared with HGPA model group; ns: no statistical difference compared with HGPA group)

Intracellular ROS levels were measured using the MitoSOX probe. The red fluorescence intensity increased significantly after HGPA stimulation, however, this effect was attenuated by NR2 (Fig. 6A, B). HGPA exposure led to elevated MDA levels and a reduction in SOD and ATP levels, which were counteracted by the high dose of NR2 (Fig. 6C). The mitochondrial potential was assessed using JC-1 staining. HGPA treatment led to a decrease in mitochondrial membrane potential, as evidenced by an increase in JC-1 monomers and a decrease in JC-1 aggregate formation. Treatment with NR2 at both medium and high doses mitigated the collapse of the mitochondrial membrane potential (Fig. 6D, E). Furthermore, Mfn2 expression was upregulated, while Drp1 and Fis1 expressions were downregulated with NR2 administration (Fig. 6F–I). These findings demonstrated that NR2 alleviated mitochondrial oxidative damage both in vivo and in vitro.

### Notoginsenoside R2 alleviated cell apoptosis

**Fig. 7:**
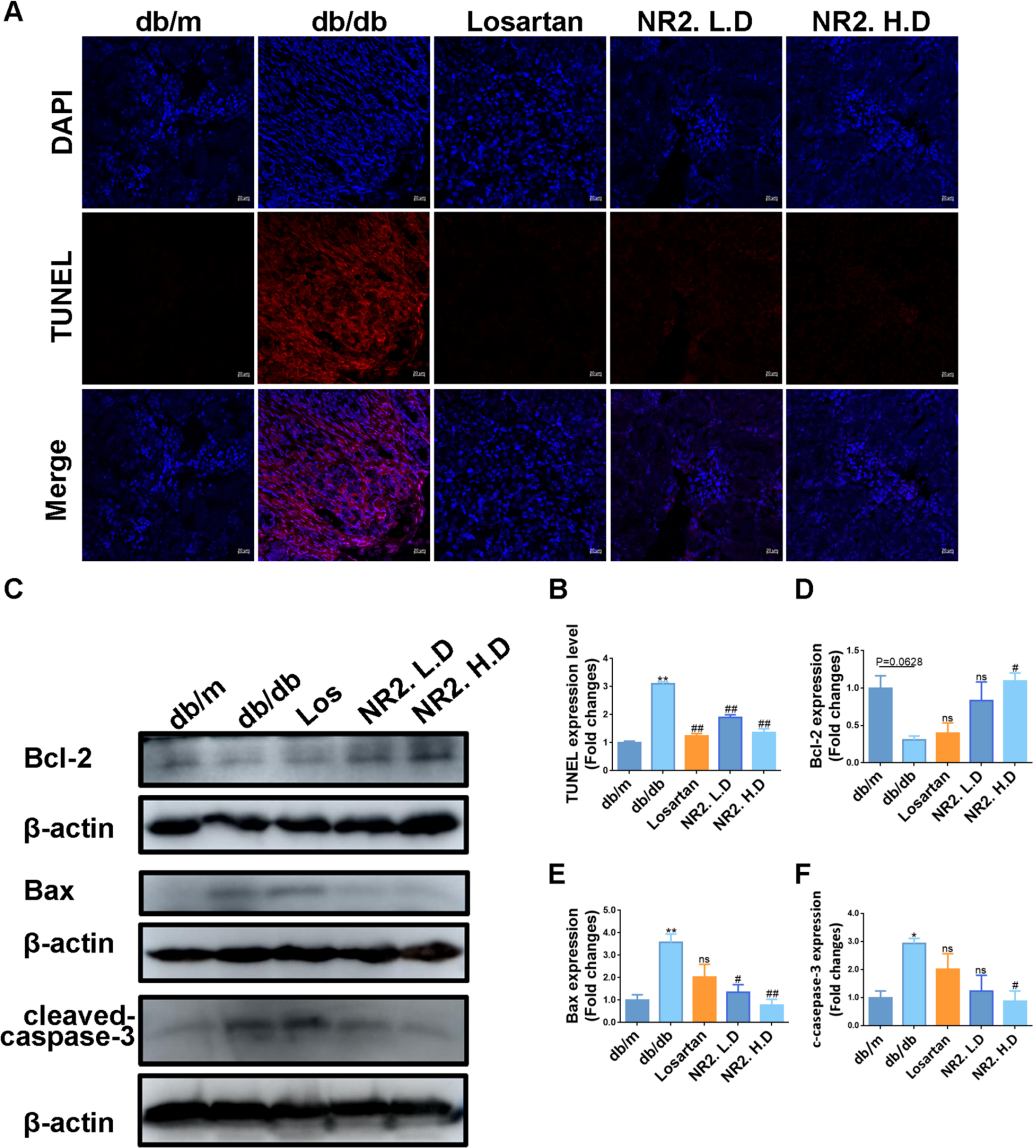
Notoginsenoside R2 alleviated cell apoptosis in db/db mice. **A**, **B** Representative images of TUNEL assay for cell apoptosis on kidney sections in each group and semiquantitative analysis (n = 3). **C**–**F** Western blot analysis for Bcl-2, Bax, and cleaved-caspase-3 protein expression in kidney tissues and quantitative analysis (n = 3). (*p < 0.05 and **p < 0.01 compared with db/m mice; ^#^p < 0.05 and ^##^p < 0.01 compared with db/db mice; ns: no statistical difference compared with db/db mice)

TUNEL staining revealed a substantial increase in TUNEL-positive cells in db/db mice, which was decreased by either NR2 or Losartan treatment (Fig. 7A, B). After NR2 treatment, the levels of pro-apoptotic proteins Bax and cleaved-caspase-3 decreased, whereas the level of the anti-apoptotic protein Bcl-2 increased, as demonstrated by WB analysis (Fig. 7C–F).

**Fig. 8:**
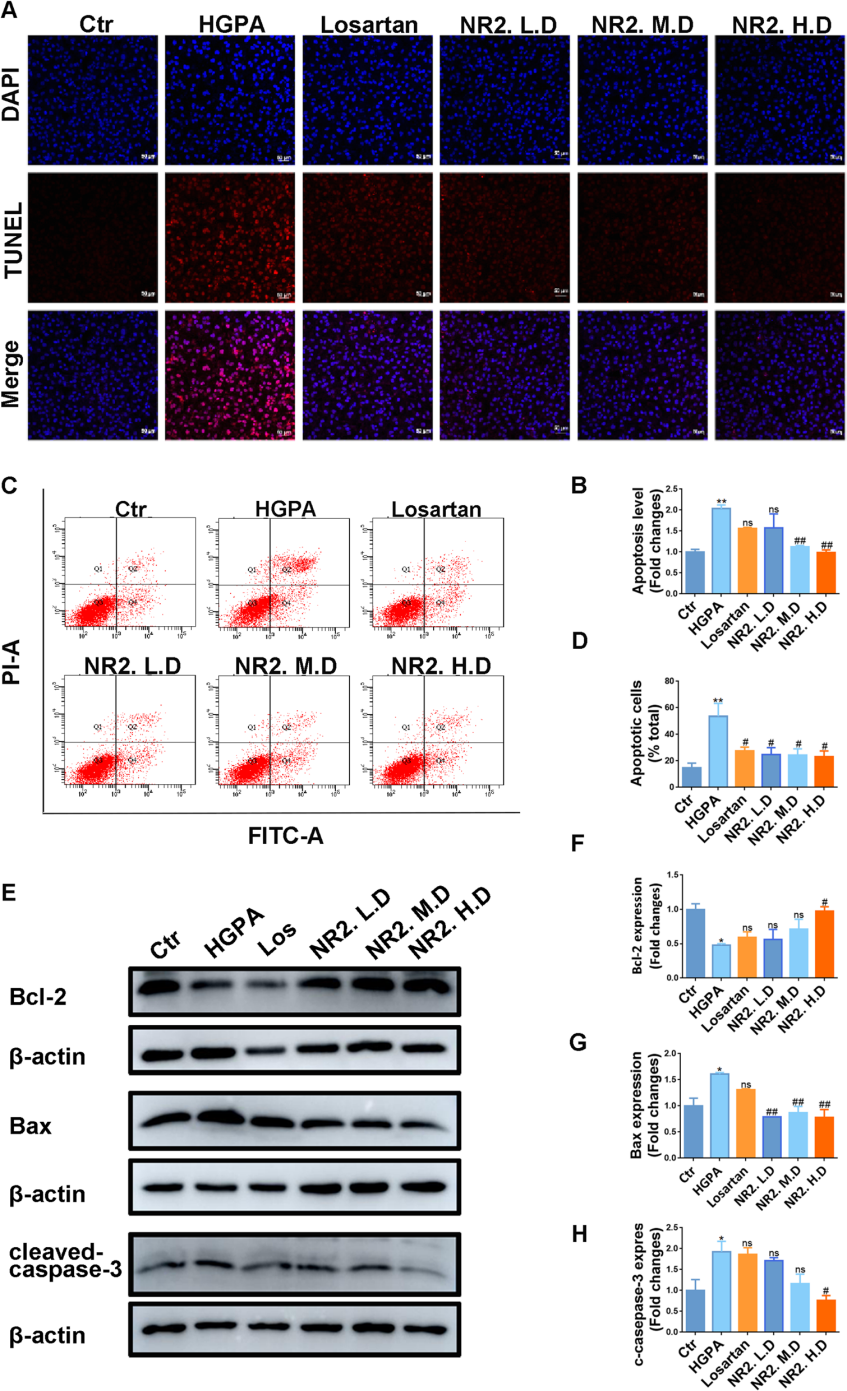
Notoginsenoside R2 alleviated cell apoptosis in HGPA-induced HK-2 cells. **A**, **B** Representative images of TUNEL assay for cell apoptosis in each group of HK-2 cells and semiquantitative analysis (n = 3). **C**, **D** Annexin V-FITC/PI staining to measure apoptotic cells by flow cytometry (n = 3). **E**–**H** Western blot analysis for Bcl-2, Bax, and cleaved-caspase-3 protein expression in HK-2 cells and quantitative analysis (n = 3). (*p < 0.05 and **p < 0.01 compared with normal control group; ^#^p < 0.05 and ^##^p < 0.01 compared with HGPA model group; ns: no statistical difference compared with HGPA group)

In vitro, HGPA stimulation caused the enhancement of TUNEL-positive cells, however, this effect was mitigated by NR2 intervention (Fig. 8A, B). Additionally, Annexin V-FITC/PI staining was employed to assess apoptotic cells, revealing that NR2 effectively attenuated HGPA-induced apoptosis in a dose-dependent manner (Fig. 8C, D). HGPA stimulation led to the upregulation of Bax and cleaved-caspase-3, while Bcl-2 expression decreased. The intervention of NR2 reversed these abnormalities (Fig. 8E–H). Taken together, these results indicated that NR2 exerted an anti-apoptotic effect.

### Inhibition of c-Src suppressed the expression of CD36 and mitigated lipid accumulation in HK-2 cells

**Fig. 9:**
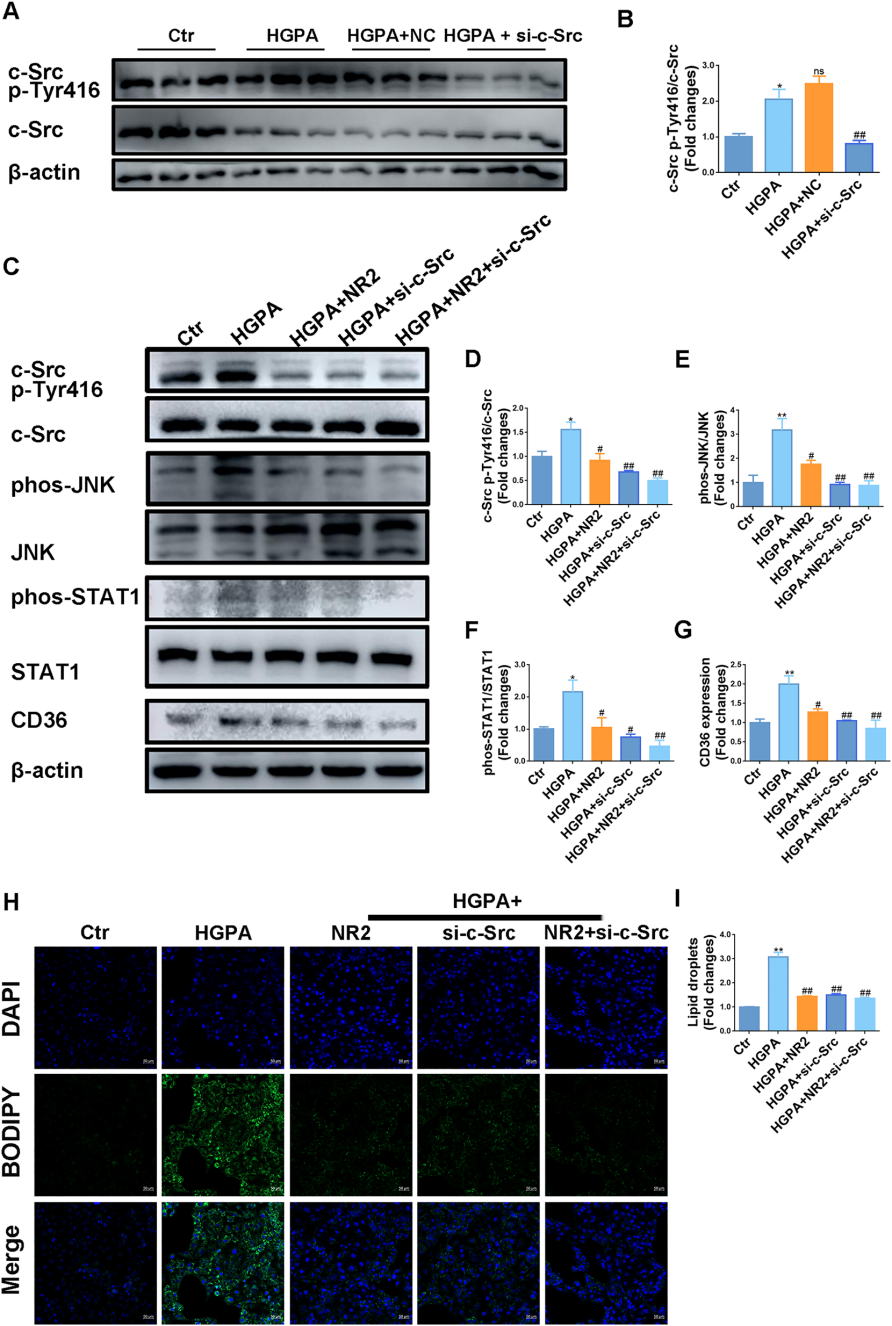
Inhibition of c-Src suppressed the expression of CD36 and mitigated lipid accumulation. **A**, **B** The expression of c-Src Tyr416 in siRNA-transfected HK-2 cells exposed to HGPA stimulation detected by Western blot analysis (n = 3). **C**–**G** Western blot analysis for c-Src Tyr416, p-JNK, p-STAT1, and CD36 in c-Src suppressed HK-2 cells exposed to HGPA stimulation and quantitative analysis (n = 3). **H**, **I** BODIPY staining to measure lipid droplets in c-Src knocked-down HK-2 cells exposed to HGPA stimulation (n = 3). (*p < 0.05 and **p < 0.01 compared with normal control group; ^#^p < 0.05 and ^##^p < 0.01 compared with HGPA model group; ns: no statistical difference compared with HGPA group)

To further validate that Notoginsenoside R2 exerted renoprotective effects by restraining c-Src, we performed c-Src knockdown using siRNA transfection. As shown in Fig. 9A, B, the upregulation of Tyr416-phosphorylated c-Src induced by HGPA was attenuated following direct c-Src inhibition. The high dose of NR2 was administered in the in vitro model, further enhancing the suppression of c-Src phosphorylation (Fig. 9C, D). The expression of p-JNK, p-STAT1 and CD36 were upregulated by HGPA stimulation, which were markedly reduced following c-Src inhibition. This improvements were further accelerated by NR2 supplementation (Fig. 9C, E–G). A remarkable reduction in BODIPY-positive cells was observed following c-Src silencing, indicating a beneficial effect on lipid accumulation (Fig. 9H, I).

### Knockdown of c-Src reversed mitochondrial dysfunction and apoptosis in HK-2 cells

**Fig. 10:**
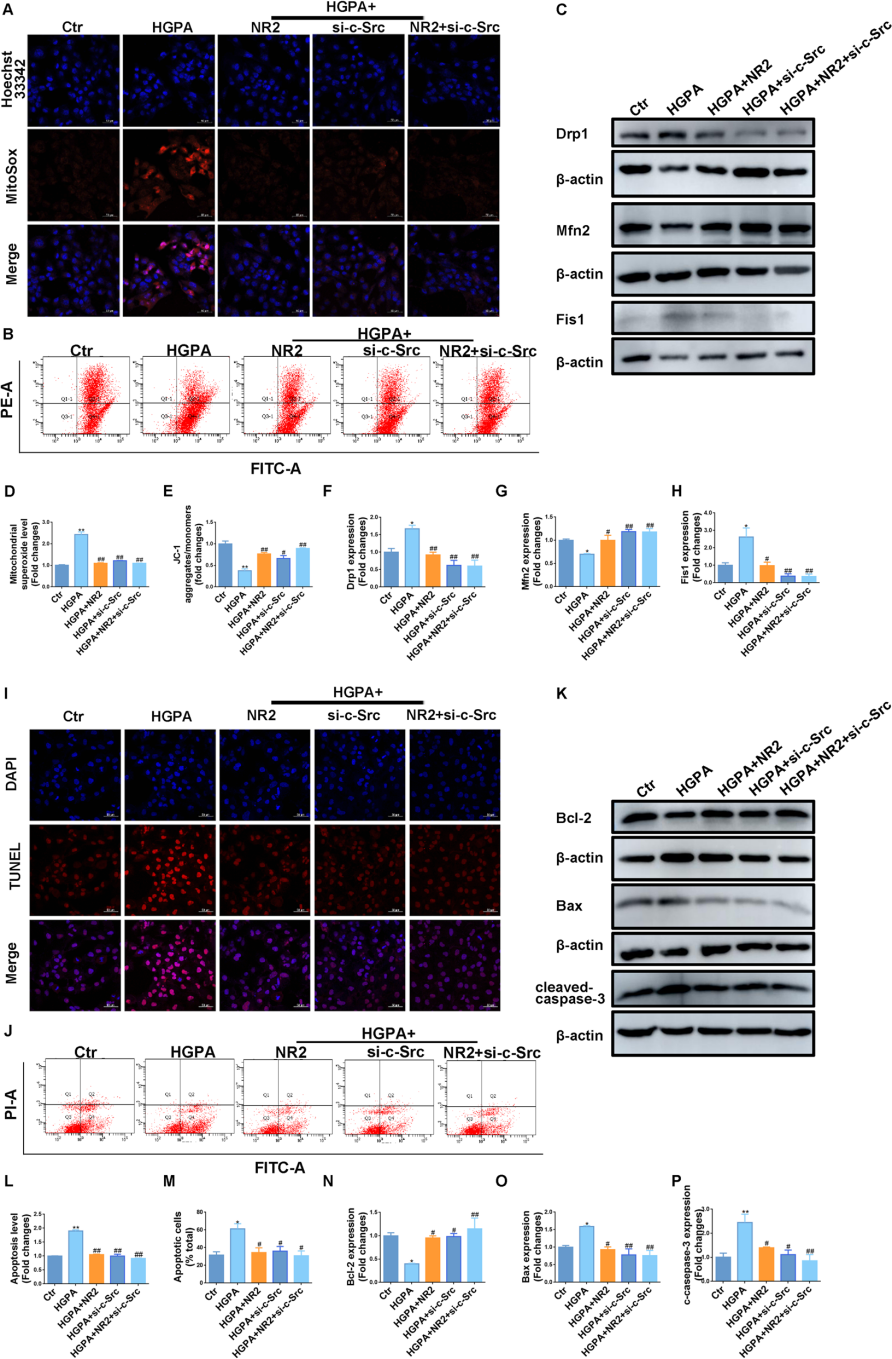
Notoginsenoside R2 reversed mitochondrial dysfunction and apoptosis by suppressing c-Src in HK-2 cells. **A** Representative images of MitoSOX dyeing and semiquantitative analysis. **B** JC-1 staining performed by flow cytometry to evaluate the mitochondrial membrane potential of each group. **C** Western blot analysis for Drp1, Mfn2, and Fis1 expression in HK-2 cells. **D** MitoSOX semiquantitative analysis (n = 3). **E** Quantitative analysis of JC-1 (n = 3). **F**–**H** Quantitative analysis of Western blot (n = 3). **I** Representative images of TUNEL assay. **J** Annexin V-FITC/PI staining to measure apoptotic cells by flow cytometry. **K** Western blot analysis for Bcl-2, Bax, and cleaved-caspase-3 expression in HK-2 cells. **L** Semiquantitative analysis of TUNEL assay (n = 3). **M** Quantitative analysis of Annexin V-FITC/PI (n = 3). **N**–**P** Quantitative analysis of Western blot (n = 3). (*p < 0.05 and **p < 0.01 compared with normal control group; ^#^p < 0.05 and ^##^p < 0.01 compared with HGPA model group; ns: no statistical difference compared with HGPA group)

HGPA stimulation resulted in elevated ROS levels. MitoSOX staining revealed that c-Src inhibition apparently decreased red fluorescence intensity, reflecting a decrease in mitochondrial ROS (Fig. 10A, D). Suppression of c-Src facilitated JC-1 aggregate formation while reducing JC-1 monomers, indicating restored mitochondrial membrane potential (Fig. 10B, E). Following c-Src inhibition, the dysregulated expression of Drp1, Mfn2, and Fis1 was normalized (Fig. 10C, F–H). These beneficial mitochondrial effects were further enhanced when combined with NR2 treatment.

TUNEL staining revealed that HGPA-induced apoptosis was attenuated by c-Src inhibition and was further enhanced with the addition of NR2 (Fig. 10I, L). Consistent findings were observed with Annexin V-FITC/PI staining, where both c-Src inhibition and NR2 treatment effectively reduced the number of apoptotic cells, with a stronger effect detected under combination therapy (Fig. 10J, M). C-Src suppression increased Bcl-2 levels while reducing Bax and cleaved-caspase-3 expression, with further improvements in these markers following NR2 co-treatment (Fig. 10K, N–P). The above findings indicated that c-Src was involved in mitochondrial dysfunction and cell apoptosis and these abnormalities could be intervened by NR2.

## Discussion

Notoginsenoside R2 is a crucial active saponin extracted from Panax notoginseng. NR2 exerted neuroprotective effects by reducing oxidative stress and inhibiting cell apoptosis [27, 28]. Our previous research demonstrated that PNS mitigated podocyte apoptosis and improved renal function [16], however, the specific benefits of NR2 for renal injury remained to be elucidated. Thus, we aimed to investigate the renoprotective effects of NR2 and clarified its underlying mechanisms in this study.

According to clinical guidelines, angiotensin receptor blockers (ARBs) are recommended for patients with both diabetes and chronic kidney disease [29]. Losartan, one of the important ARBs, has demonstrated renoprotective benefits in patients with nephropathy, including those with both type 1 and type 2 diabetes [30]. Previous study demonstrated that the dosage of Losartan (10 mg/kg) ameliorated hypertension, albuminuria and renal histological parameters in db/db mice [31]. Additionally, losartan (10 mg/kg) served as a positive control in our previous study [22]. In this study, we selected losartan (10 mg/kg) as the positive drug to evaluate the therapeutic effects of NR2 on DN.

Both Notoginsenoside R1 and NR2 are saponins isolated from Chinese herb Panax notoginseng. We previously reported that Notoginsenoside R1 ameliorated albuminuria and histopathology in diabetic animals [17]. Previous study further demonstrated that 30 mg/kg/day of Notoginsenoside R1 ameliorated urinary albumin and renal histological abnormalities in db/db mice [32].The dosage of 20 mg/kg/day of NR2 was orally given to the mice [23]. The dosages of NR2 in this study were selected according to the published literature and our preliminary experiment.

Our findings demonstrated that NR2 prevented the progression of DN partly through restraining the activation of c-Src, thereby reducing CD36 overexpression and improving lipid metabolism. The following evidence supported our conclusion: (1) NR2 treatment ameliorated albuminuria, renal dysfunction, and structural abnormalities. (2) In both in vivo and in vitro models, NR2 inhibited Tyr416 phosphorylation of c-Src and downregulated CD36 expression, while also suppressing the JNK/STAT1 pathway, an upstream regulator of CD36. (3) NR2 reduced lipid droplets, mitigated mitochondrial dysfunction and cell apoptosis in db/db mice and HGPA-induced HK-2 cells. (4) C-Src inhibition alone was sufficient to decrease CD36 expression and alleviate mitochondrial and apoptotic impairments, with these effects further enhanced by NR2. Therefore, NR2 might be a therapeutic agent for DN management.

The main Src family kinase (SFK), c-Src, interacts with various proteins and protein complexes, functioning as a non-receptor tyrosine kinase necessary for signal transduction. The activation of c-Src is primarily regulated by phosphorylation at two key tyrosine residues: Tyr416 and Tyr527. Under normal physiological conditions, c-Src remains inactive, with activation initiated by phosphorylation of Tyr416. Previous studies reported c-Src activation in DN models, where its inhibition was associated with reduced albuminuria and the mitigation of renal histopathological damage [33, 34]. In our study, we observed significant c-Src phosphorylation at Tyr416 in both in vivo (db/db mice) and in vitro (HGPA-induced HK-2 cells) DN models and this phosphorylation was effectively suppressed by NR2 treatment. These findings suggested that the renoprotective effects exhibited by NR2 were correlated with the inactivation of c-Src.

Healthy kidneys have a lipid content of less than 3% of their wet weight [35]. Animal experiments and clinical studies showed that lipids directly influence the onset and progression of renal disorders [36]. The inhibition of CD36 relieved lipid accumulation and improved podocyte dysfunction in obesity-related glomerulopathy [37]. In our study, we noted a significant reduction in lipid droplets and CD36 expression after NR2 intervention in both in vivo and in vitro models. Additionally, c-Src inhibition attenuated lipid accumulation and downregulated CD36 expression in HGPA-stimulated HK-2 cells, alongside reduced phosphorylation of JNK/STAT1, an upstream regulatory pathway of CD36.

An imbalance between oxidants and antioxidants is a primary driver of oxidative stress, which causes excessive ROS production that damages cells and tissues [38]. Superfluous free fatty acids were shown to trigger excessive ROS. Research indicated that exposure to palmitic acid (PA) gave rise to significant ROS generation in the cytoplasm and mitochondria of podocytes, resulting in mitochondrial membrane depolarization, decreased ATP synthesis, and apoptosis [39]. Similar results were observed in experiments with HK-2 cells [40]. Mitochondrial oxidative damage, in turn, further resulted in the reprogramming of lipid metabolism, leading to lipid droplet formation, impaired fatty acid oxidation, and lipid peroxidation, all of which exacerbate the progression of DN [14]. Antioxidants lowered ROS levels and mitigate oxidative stress, thereby reversing lipid metabolism disorders and reducing lipid accumulation [41]. CD36 features a positively charged domain that binds negatively charged ligands, such as oxidized HDL, oxidized phospholipids and advanced glycation end-products [35]. This ligand-binding versatility makes CD36 a critical target for ameliorating cellular metabolic dysfunction. The use of selective CD36 inhibitors and mitochondrial antioxidants could reduce cellular damage induced by PA [39]. Inhibiting CD36 binding sites reduced PA-induced lipid accumulation and ROS production [9]. Beyond reversing renal dysfunction, glomerular hypertrophy, and tubulointerstitial fibrosis, CD36 depletion showed protective effects on mitochondrial function by restoring mitochondrial morphology and fatty acid oxidation [42].

In our study, we observed exacerbated oxidative stress and disturbances in mitochondrial dynamics in the model group. Specifically, the model group exhibited increased levels of the lipid peroxide MDA and decreased levels of redox capacity markers. Additionally, the expression of Mfn2 was downregulated, while the fission proteins Drp1 and Fis1 were significantly upregulated. Notably, NR2 treatment effectively restored these aberrant expression patterns, mitigated oxidative stress, and enhanced mitochondrial function. In the in vitro model, oxidative stress was significantly elevated, as evidenced by elevated mitochondrial superoxide levels, collapsed mitochondrial membrane potential, and dysregulated expression of proteins related to mitochondrial dynamics. These perturbations were ameliorated by NR2 intervention. Additionally, the aforementioned aberrant conditions were alleviated by c-Src knockdown. Finally, our study demonstrated that NR2 intervention reversed cell apoptosis, a downstream consequence of mitochondrial dysfunction.

Our study clearly demonstrated the protective effects of NR2 on DN and explored its underlying mechanisms. We previously reported that Notoginsenoside R1 improved podocyte adhesion under diabetic conditions by upregulating α3β1 integrin [17] and Notoginsenoside Fc reduced glomerular endothelial cell pyroptosis by regulating HMGCS2 expression [18]. In this study, we observed that NR2 exerted renoprotective effects in DN through different mechanisms. Ginsenoside Rg1 was reported to inhibit CD36 overexpression and improve lipid metabolism, probably through direct antagonistic interaction with CD36, as supported by molecular docking analysis [20]. Our findings indicated that NR2 downregulated CD36 expression by targeting c-Src. These novel findings suggested differential effects among NR2 and other saponins of PNS and highlighted c-Src as a promising therapeutic target for DN.

Our results also suggested that restoring lipid metabolism might represent a promising therapeutic strategy for DN. Metabolic reprogramming, wherein cells adjust their metabolic state to satisfy energy demands for survival and growth, has been linked to DN. Impaired mitochondrial biosynthesis dysfunction could trigger metabolic reprogramming in diabetic conditions that is closely associated with DN progression [43]. Metabolic disorders could induce oxidative stress, inflammatory damage, and apoptosis, which contributed to DN development and resulted in renal fibrosis [44]. Previous research suggested that mitochondrial oxidative stress might influence lipid metabolism-driven reprogramming of tubular epithelial cells during DN progression. Restoring dysregulated lipid metabolism to protect mitochondrial function might offer a novel therapeutic approach for DN [14]. In macrophages, CD36 was associated with dysregulated lipid metabolism and mitochondrial oxidative stress, promoting chronic inflammation [45]. In view of the ameliorative effects of NR2 in reducing lipid deposition and CD36 expression, NR2 might serve as a potential strategy for reversing metabolic reprogramming in DN, which will be further examined in future studies. Lipid metabolism is a complex process and the abnormal accumulation of TG is often associated with disrupted phospholipid homeostasis. The balance between phospholipids, particularly phosphatidylethanolamine, plays a crucial role in maintaining lipid homeostasis [46]. Whether NR2 could restore lipid homeostasis remains to be determined in the future study.

Finally, the toxicity of NR2 on the major organs was examined. The effects of NR2 on liver and kidney function were assessed by measuring blood ALT, AST, Scr, and BUN levels. The results showed that NR2 did not alter the levels of these biomarkers, suggesting that NR2 did not exhibit apparent toxicity to the liver or kidney.

Our study showed the beneficial effects of NR2 on DN for the first time. However, further research is required before its practical application. Combining small molecule with nano-coordinated polymer particles could enhance the oral bioavailability and intensify the effectiveness [47]. These studies are particularly helpful for the future investigations of NR2.

NR2 is one of the components of PNS. Previous study demonstrated that PNS improved the pathological changes of the thymus and spleen in rats with with severe acute pancreatitis [48]. Fermented Notoginseng saponins decreased the enlargement of spleen and thymus atrophy and exerted protective effects on immune organs in blood deficiency rats [49]. PNS decreased M1 macrophages in spleen and colon tissues in rats with dextran sulfate sodium-induced colitis [50]. These studies suggested that PNS might have protective effect on the spleen and immune organs.

However, there were limitations in our study. Firstly, the effects of NR2 on the spleen and immune system should be further investigated examined in the future. Secondly, the effects of NR2 on fatty acid absorption, fatty acid oxidation and cholesterol metabolism should be examined in future studies.

## Conclusion

**Fig. 11:**
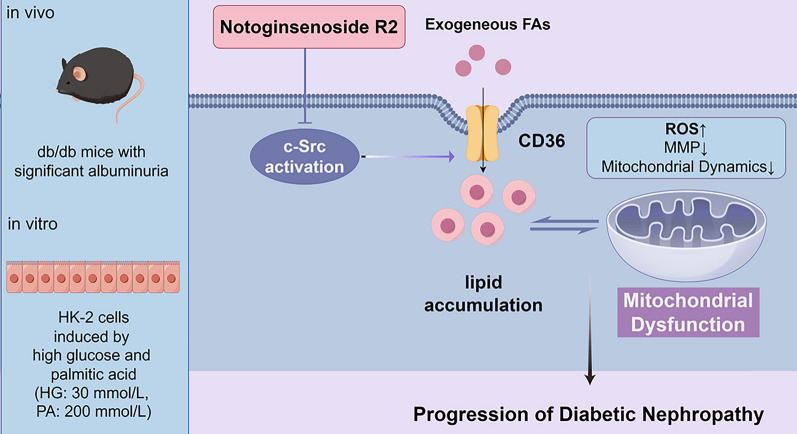
The Schematic illustration of the mechanism of Notoginsenoside R2 preventing DN progression

In summary, our findings clearly demonstrated that NR2 ameliorated mitochondrial dysfunction and delayed the progression of DN partly by c-Src suppression. The beneficial effects of NR2 might be involved in the reduction of lipid accumulation (Fig. 11). These novel findings might provide a potential novel therapeutic strategy for DN.

## Data Availability

Information about research data will be available upon request.

## Abbreviations

ACR: Albumin-to-creatinine ratios
ALT: Alanine transaminase
AST: Aspartate transaminase
BUN: Blood urea nitrogen
CAT: Catalase
CD36: Cluster of differentiation 36
CKD: Chronic kidney disease
DN: Diabetic nephropathy
Drp-1: Dynamin-related protein 1
ESRD: End-stage renal disease
Fis1: Fission1
GR: Glutathione reductase
GSH: Glutathione
HE: Hematoxylin and eosin
HGPA: High glucose and palmitic acid
IHC: Immunohistochemistry
JNK: C-Jun N-terminal kinase
MDA: Malondialdehyde
Mfn2: Mitofusion 2
NR2: Notoginsenoside R2
PA: Palmitic acid
PAS: Periodic acid-Schiff
PNS: Panax notoginseng saponins
ROS: Reactive oxygen species
Scr: Serum creatinine
STAT1: Signal transducerand activator of transcription 1
SOD: Superoxide dismutase
TC: Total cholesterol
TG: Triglyceride
TEM: Transmission electron microscopy
WB: Western blot

## References

[1] Kato M, Natarajan R. Epigenetics and epigenomics in diabetic kidney disease and metabolic memory. Nat Rev Nephrol. 2019;15(6):327–45. 30894700 10.1038/s41581-019-0135-6 PMC6889804

[2] Couser WG, Remuzzi G, Mendis S, Tonelli M. The contribution of chronic kidney disease to the global burden of major noncommunicable diseases. Kidney Int. 2011;80(12):1258–70. 21993585 10.1038/ki.2011.368

[3] Opazo-Ríos L, Mas S, Marín-Royo G, Mezzano S, Gómez-Guerrero C, Moreno JA, Egido J. Lipotoxicity and diabetic nephropathy: novel mechanistic insights and therapeutic opportunities. Int J Mol Sci. 2020;21(7):2632. 32290082 10.3390/ijms21072632 PMC7177360

[4] Chen SC, Tseng CH. Dyslipidemia, kidney disease, and cardiovascular disease in diabetic patients. Rev Diabet Stud. 2013;15(6):327–45. 10.1900/RDS.2013.10.88 PMC4063094 24380085

[5] Coburn CT, Knapp FF Jr, Febbraio M, Beets AL, Silverstein RL, Abumrad NA. Defective uptake and utilization of long chain fatty acids in muscle and adipose tissue of CD36 knockout mice. J Biol Chem. 2000;275(42):32523–9. 10913136 10.1074/jbc.M003826200

[6] Glatz JF, Luiken JJ. From fat to FAT (CD36/SR-B2): understanding the regulation of cellular fatty acid uptake. Biochimie. 2017;136:21–6. 28013071 10.1016/j.biochi.2016.12.007

[7] Feng L, Gu C, Li Y, Huang J. High glucose promotes CD36 expression by upregulating peroxisome proliferator-activated receptor γ levels to exacerbate lipid deposition in renal tubular cells. Biomed Res Int. 2017;2017:1414070. 28497039 10.1155/2017/1414070 PMC5405368

[8] Bessi VL, Labbe SM, Huynh DN, Menard L, Jossart C, Febbraio M, et al. EP 80317, a selective CD36 ligand, shows cardioprotective effects against post-ischaemic myocardial damage in mice. Cardiovasc Res. 2012;96:99–108. 22787133 10.1093/cvr/cvs225

[9] Hua W, Huang HZ, Tan LT, Wan JM, Gui HB, Zhao L, et al. CD36 mediated fatty acid-induced podocyte apoptosis via oxidative stress. PLoS ONE. 2015;10(5):e0127507. 26000608 10.1371/journal.pone.0127507 PMC4441449

[10] Kang HM, Ahn SH, Choi P, Ko YA, Han SH, Chinga F, Park AS, Tao J, Sharma K, Pullman J, Bottinger EP, Goldberg IJ, Susztak K. Defective fatty acid oxidation in renal tubular epithelial cells has a key role in kidney fibrosis development. Nat Med. 2015;21(1):37–46. 25419705 10.1038/nm.3762 PMC4444078

[11] Linley JE, Ooi L, Pettinger L, Kirton H, Boyle JP, Peers C, Gamper N. Reactive oxygen species are second messengers of neurokinin signaling in peripheral sensory neurons. Proc Natl Acad Sci USA. 2012;109(24):E1578–86. 22586118 10.1073/pnas.1201544109 PMC3386117

[12] Shi T, Dansen TB. Reactive oxygen species induced p53 activation: DNA damage, redox signaling, or both? Antioxid Redox Signal. 2020;33(12):839–59. 32151151 10.1089/ars.2020.8074

[13] Xu M, Wang W, Cheng J, Qu H, Xu M, Wang L. Effects of mitochondrial dysfunction on cellular function: role in atherosclerosis. Biomed Pharmacother. 2024;174:116587. 38636397 10.1016/j.biopha.2024.116587

[14] Hou Y, Tan E, Shi H, Ren X, Wan X, Wu W, et al. Mitochondrial oxidative damage reprograms lipid metabolism of renal tubular epithelial cells in the diabetic kidney. Cell Mol Life Sci. 2024;81(1):23. 38200266 10.1007/s00018-023-05078-y PMC10781825

[15] Mi W, Yu M, Yin S, Ji Y, Shi T, Li N. Analysis of the renal protection and antioxidative stress effects of *Panax notoginseng* saponins in diabetic nephropathy mice. J Immunol Res. 2022;2022:3610935. 36249425 10.1155/2022/3610935 PMC9556179

[16] Xue R, Zhai R, Xie L, Zheng Z, Jian G, Chen T, et al. Xuesaitong protects podocytes from apoptosis in diabetic rats through modulating PTEN-PDK1-Akt-mTOR pathway. J Diabetes Res. 2020;2020:9309768. 32051833 10.1155/2020/9309768 PMC6995497

[17] Gui D, Wei L, Jian G, Guo Y, Yang J, Wang N. Notoginsenoside R1 ameliorates podocyte adhesion under diabetic condition through α3β1 integrin upregulation *in vitro* and *in vivo*. Cell Physiol Biochem. 2014;34(6):1849–62. 25503068 10.1159/000366384

[18] Shen Y, Chen W, Lin K, Zhang H, Guo X, An X, et al. Notoginsenoside Fc, a novel renoprotective agent, ameliorates glomerular endothelial cells pyroptosis and mitochondrial dysfunction in diabetic nephropathy through regulating HMGCS2 pathway. Phytomedicine. 2024;126:155445. 38412666 10.1016/j.phymed.2024.155445

[19] Liu H, Chen W, Lu P, Ma Y, Liang X, Liu Y. Ginsenoside Rg1 attenuates the inflammation and oxidative stress induced by diabetic nephropathy through regulating the PI3K/AKT/FOXO3 pathway. Ann Transl Med. 2021;9(24):1789. 35071483 10.21037/atm-21-6234 PMC8756242

[20] Han Y, Su Y, Han M, Liu Y, Shi Q, Li X, et al. Ginsenoside Rg1 attenuates glomerular fibrosis by inhibiting CD36/TRPC6/NFAT2 signaling in type 2 diabetes mellitus mice. J Ethnopharmacol. 2023;302(Pt A):115923. 36375645 10.1016/j.jep.2022.115923

[21] Jiang Y, Sui D, Li M, Xu H, Yu X, Liu J, et al. Ginsenoside Re improves inflammation and fibrosis in hepatic tissue by upregulating PPARγ expression and inhibiting oxidative stress in db/db mice. Evid Based Complement Alternat Med. 2021;2021:9003603. 34659439 10.1155/2021/9003603 PMC8519719

[22] Dong R, Zhang X, Liu Y, Zhao T, Sun Z, Liu P, et al. Rutin alleviates EndMT by restoring autophagy through inhibiting HDAC1 via PI3K/AKT/mTOR pathway in diabetic kidney disease. Phytomedicine. 2023;112:154700. 36774842 10.1016/j.phymed.2023.154700

[23] Li X, Zhang Y, Hong Z, Gong S, Liu W, Zhou X, et al. Transcriptome profiling analysis reveals the potential mechanisms of three bioactive ingredients of Fufang E’jiao Jiang during chemotherapy-induced myelosuppression in mice. Front Pharmacol. 2018;13(9):616. 10.3389/fphar.2018.00616 PMC6008481 29950993

[24] Wu L, Liu C, Chang DY, Zhan R, Zhao M, Lam SM, et al. The attenuation of diabetic nephropathy by annexin A1 via regulation of lipid metabolism through the AMPK/PPARalpha/CPT1b pathway. Diabetes. 2021;70(10):2192–203. 34103347 10.2337/db21-0050

[25] Liu Y, Zhong Y, Chen H, Wang D, Wang M, Ou JS, et al. Retinol binding protein-dependent cholesterol uptake regulates macrophage foam cell formation and promotes atherosclerosis. Circulation. 2017;135(14):1339–54. 28122883 10.1161/CIRCULATIONAHA.116.024503

[26] Gai Z, Wang T, Visentin M, Kullak-Ublick GA, Fu X, Wang Z. Lipid accumulation and chronic kidney disease. Nutrients. 2019;11(4):722. 10.3390/nu11040722 PMC6520701 30925738

[27] Meng XB, Sun GB, Wang M, Sun J, Qin M, Sun XB. P90RSK and Nrf2 activation via MEK1/2-ERK1/2 pathways mediated by notoginsenoside R2 to prevent 6-hydroxydopamine-induced apoptotic death in SH-SY5Y cells. Evid Based Complement Alternat Med. 2013;2013:971712. 10.1155/2013/971712 PMC3789498 24159358

[28] Hu Y, Wu L, Jiang L, Liang N, Zhu X, He Q, et al. Notoginsenoside R2 reduces Aβ25–35-induced neuronal apoptosis and inflammation via miR-27a/SOX8/β-catenin axis. Hum Exp Toxicol. 2021;40(12):S347–58. 34533063 10.1177/09603271211041996

[29] Kidney Disease: Improving Global Outcomes (KDIGO) Diabetes Work Group. KDIGO 2022 clinical practice guideline for diabetes management in chronic kidney disease. Kidney Int. 2022;102(5S):S1–127. 36272764 10.1016/j.kint.2022.06.008

[30] Brenner BM, Cooper ME, de Zeeuw D, Keane WF, Mitch WE, Parving HH, et al. Effects of losartan on renal and cardiovascular outcomes in patients with type 2 diabetes and nephropathy. N Engl J Med. 2001;345(12):861–9. 11565518 10.1056/NEJMoa011161

[31] Reddy MA, Sumanth P, Lanting L, Yuan H, Wang M, Mar D, et al. Losartan reverses permissive epigenetic changes in renal glomeruli of diabetic db/db mice. Kidney Int. 2014;85(2):362–73. 24088954 10.1038/ki.2013.387 PMC3946617

[32] Zhang B, Zhang X, Zhang C, Shen Q, Sun G, Sun X. Notoginsenoside R1 protects db/db mice against diabetic nephropathy via upregulation of Nrf2-mediated HO-1 expression. Molecules. 2019;24(2):247. 30634720 10.3390/molecules24020247 PMC6359411

[33] Wang J, Zhuang S. Src family kinases in chronic kidney disease. Am J Physiol Renal Physiol. 2017;313(3):F721–8. 28615246 10.1152/ajprenal.00141.2017 PMC5625110

[34] Taniguchi K, Xia L, Goldberg HJ, Lee KW, Shah A, Stavar L, et al. Inhibition of Src kinase blocks high glucose-induced EGFR transactivation and collagen synthesis in mesangial cells and prevents diabetic nephropathy in mice. Diabetes. 2013;62(11):3874–86. 23942551 10.2337/db12-1010 PMC3806624

[35] Yang X, Okamura DM, Lu X, Chen Y, Moorhead J, Varghese Z, et al. CD36 in chronic kidney disease: novel insights and therapeutic opportunities. Nat Rev Nephrol. 2017;13(12):769–81. 28919632 10.1038/nrneph.2017.126

[36] de Vries AP, Ruggenenti P, Ruan XZ, Praga M, Cruzado JM, Bajema IM, et al. Fatty kidney: emerging role of ectopic lipid in obesity-related renal disease. Lancet Diabetes Endocrinol. 2014;2(5):417–26. 24795255 10.1016/S2213-8587(14)70065-8

[37] Zhao J, Rui HL, Yang M, Sun LJ, Dong HR, Cheng H. CD36-mediated lipid accumulation and activation of NLRP3 inflammasome lead to podocyte injury in obesity-related glomerulopathy. Mediators Inflamm. 2019;2019:3172647. 10.1155/2019/3172647 PMC6487104 31097920

[38] Kao MP, Ang DS, Pall A, Struthers AD. Oxidative stress in renal dysfunction: mechanisms, clinical sequelae and therapeutic options. J Hum Hypertens. 2010;24(1):1–8. 19727125 10.1038/jhh.2009.70

[39] Xu S, Nam SM, Kim JH, Das R, Choi SK, Nguyen TT, et al. Palmitate induces ER calcium depletion and apoptosis in mouse podocytes subsequent to mitochondrial oxidative stress. Cell Death Dis. 2015;6(11):e1976. 26583319 10.1038/cddis.2015.331 PMC4670935

[40] Chen Q, Su Y, Ju Y, Ma K, Li W, Li W. Astragalosides IV protected the renal tubular epithelial cells from free fatty acids-induced injury by reducing oxidative stress and apoptosis. Biomed Pharmacother. 2018;108:679–86. 30245468 10.1016/j.biopha.2018.09.049

[41] Shen Y, Huang H, Wang Y, Yang R, Ke X. Antioxidant effects of Se-glutathione peroxidase in alcoholic liver disease. J Trace Elem Med Biol. 2022;74:127048. 35963055 10.1016/j.jtemb.2022.127048

[42] Niu H, Ren X, Tan E, Wan X, Wang Y, Shi H, et al. CD36 deletion ameliorates diabetic kidney disease by restoring fatty acid oxidation and improving mitochondrial function. Ren Fail. 2023;45(2):2292753. 38097943 10.1080/0886022X.2023.2292753 PMC10732185

[43] Wang M, Pang Y, Guo Y, Tian L, Liu Y, Shen C, et al. Metabolic reprogramming: a novel therapeutic target in diabetic kidney disease. Front Pharmacol. 2022;13:970601. 36120335 10.3389/fphar.2022.970601 PMC9479190

[44] Fan X, Yang M, Lang Y, Lu S, Kong Z, Gao Y, et al. Mitochondrial metabolic reprogramming in diabetic kidney disease. Cell Death Dis. 2024;15(6):442. 10.1038/s41419-024-06833-0 PMC11194272 38910210

[45] Chen Y, Yang M, Huang W, Chen W, Zhao Y, Schulte ML, et al. Mitochondrial metabolic reprogramming by CD36 signaling drives macrophage inflammatory responses. Circ Res. 2019;125(12):1087–102. 31625810 10.1161/CIRCRESAHA.119.315833 PMC6921463

[46] Chen Y, Jiang H, Zhan Z, Lu J, Gu T, Yu P, et al. Oridonin restores hepatic lipid homeostasis in an LXRα-ATGL/EPT1 axis-dependent manner. J Pharm Anal. 2023;13(11):1281–95. 10.1016/j.jpha.2023.08.010 PMC10759262 38174118

[47] Gui SY, Wang XC, Huang ZH, Li MM, Wang JH, Gui SY, et al. Nanoscale coordination polymer Fe-DMY downregulates the Poldip2-Nox4-H_2_O_2_ pathway and alleviates diabetic retinopathy. J Pharm Anal. 2023;13(11):1326–45. 38174114 10.1016/j.jpha.2023.05.002 PMC10759264

[48] Zhang XP, Jiang J, Cheng QH, Ye Q, Li WJ, et al. Protective effects of Ligustrazine, Kakonein and Panax Notoginsenoside on the small intestine and immune organs of rats with severe acute pancreatitis. Hepatobiliary Pancreat Dis Int. 2011;10(6):632–7. 22146628 10.1016/s1499-3872(11)60107-0

[49] Su W, Liang Z, Pan D, Zhang L, Zhang Y, Yuan T, et al. Therapeutic effect of notoginseng saponins before and after fermentation on blood deficiency rats. Exp Ther Med. 2024;27(4):143. 38476921 10.3892/etm.2024.12431 PMC10928825

[50] Lu QG, Zeng L, Li XH, Liu Y, Du XF, Bai GM, et al. Protective effects of panax notoginseng saponin on dextran sulfate sodium-induced colitis in rats through phosphoinositide-3-kinase protein kinase B signaling pathway inhibition. World J Gastroenterol. 2020;26(11):1156–71. 32231420 10.3748/wjg.v26.i11.1156 PMC7093311

